# Validation of high temporal resolution spiral phase velocity mapping of temporal patterns of left and right coronary artery blood flow against Doppler guidewire

**DOI:** 10.1186/s12968-015-0189-y

**Published:** 2015-10-02

**Authors:** Jennifer Keegan, Claire E Raphael, Kim Parker, Robin M Simpson, Stephen Strain, Ranil de Silva, Carlo Di Mario, Julian Collinson, Rod H Stables, Ricardo Wage, Peter Drivas, Malindie Sugathapala, Sanjay K Prasad, David N Firmin

**Affiliations:** Cardiovascular Biomedical Research Unit, Royal Brompton and Harefield NHS Foundation Trust, London, UK; Department of Bioengineering, Imperial College London, London, UK; Radiological Physics, University Medical Centre, Freiburg, Germany; National Heart and Lung Institute, Imperial College London, London, UK; Department of Cardiology, Royal Brompton and Harefield NHS Foundation Trust, London, UK; Department of Cardiology, Chelsea and Westminster Hospital, London, UK; Institue of Cardiovascular Science and Medicine, Liverpool Heart and Chest Hospital, Liverpool, UK

**Keywords:** Spiral, Phase velocity mapping, Coronary blood flow, Temporal pattern, Doppler, Validation

## Abstract

**Background:**

Temporal patterns of coronary blood flow velocity can provide important information on disease state and are currently assessed invasively using a Doppler guidewire. A non-invasive alternative would be beneficial as it would allow study of a wider patient population and serial scanning.

**Methods:**

A retrospectively-gated breath-hold spiral phase velocity mapping sequence (TR 19 ms) was developed at 3 Tesla. Velocity maps were acquired in 8 proximal right and 15 proximal left coronary arteries of 18 subjects who had previously had a Doppler guidewire study at the time of coronary angiography. Cardiovascular magnetic resonance (CMR) velocity-time curves were processed semi-automatically and compared with corresponding invasive Doppler data.

**Results:**

When corrected for differences in heart rate between the two studies, CMR mean velocity through the cardiac cycle, peak systolic velocity (PSV) and peak diastolic velocity (PDV) were approximately 40 % of the peak Doppler values with a moderate - good linear relationship between the two techniques (R^2^: 0.57, 0.64 and 0.79 respectively). CMR values of PDV/PSV showed a strong linear relationship with Doppler values with a slope close to unity (0.89 and 0.90 for right and left arteries respectively). In individual vessels, plots of CMR velocities at all cardiac phases against corresponding Doppler velocities showed a consistent linear relationship between the two with high R^2^ values (mean +/−SD: 0.79 +/−.13).

**Conclusions:**

High temporal resolution breath-hold spiral phase velocity mapping underestimates absolute values of coronary flow velocity but allows accurate assessment of the temporal patterns of blood flow.

## Background

While blood flow in the majority of arteries peaks in systole, the rhythmic squeezing of the intramyocardial arterioles and microcirculation as the heart beats results in left anterior descending (LAD) artery flow being diastolic-predominant while for the right coronary artery (RCA), there is approximately equal flow in systole and diastole [1, 2]. These temporal flow patterns are affected by disease and can provide important information on disease state [3–5] and on the results of interventional procedures [6]. The ‘gold standard’ for coronary artery flow velocity assessment is the Doppler guide wire which is inserted directly into the artery under X-Ray fluoroscopic guidance [7]. However, the radiation dose involved and the small but significant risk of complications effectively limit the use of the technique to clinical studies and longitudinal research studies are unlikely to be approved by ethics bodies or be attractive to patients. A non-invasive method of obtaining these flow patterns would be highly beneficial.

Cardiovascular magnetic resonance (CMR) measurements of coronary artery blood flow have generally been performed using a breath-hold segmented gradient echo phase velocity mapping technique [8–11] but there are few direct comparisons with Doppler guidewire studies. An early study showed that CMR measures of absolute coronary flow agreed well with Doppler guidewire assessed values but these CMR data were acquired with only 4 or 5 cine frames per cardiac cycle and provided no information on the temporal flow pattern [12]. The subsequent implementation of view-sharing techniques allowed the number of cine frames to be increased to 9–13 [13] and using this approach, it was shown that while CMR measures of peak flow velocity in the cardiac cycle are significantly less than those measured by Doppler, there was a good linear relationship between the techniques when assessing the increase in peak diastolic velocity in response to dipyridamole (the coronary flow velocity reserve), the coefficient of determination (R^2^) being 0.83 [14]. A further study comparing breath-hold and navigator-gated CMR acquisitions with Doppler data showed that on average, the peak diastolic CMR velocities (averaged over the vessel) were 0.33 and 0.37 respectively of the corresponding peak Doppler velocities but that the correlations between them were moderately good (R^2^ values of 0.49 and 0.74 respectively) [15]. The higher correlation observed in the navigator-gated study reflects the improved temporal resolution of the technique which had an acquisition window of 45 ms compared to 140 ms for the breath-hold acquisition.

The high in-plane motion of the coronary arteries through the cardiac cycle [16] imposes limitations on the acquisition window of coronary phase velocity mapping studies, estimated at 58 ms for the left anterior descending artery and 23 ms for the more mobile right coronary artery [17]. It is not feasible to achieve either of these using breath-hold segmented gradient echo phase velocity mapping and while navigator gated free breathing could be used, the acquisition durations would be long and unpredictable. Spiral k-space coverage is more efficient than Cartesian coverage and enables shorter acquisition windows and a higher temporal resolution within a breath-hold duration. Early studies have demonstrated the advantages of spiral over Cartesian coronary flow acquisitions, particularly for the highly mobile right coronary artery [18–20]. More recently, such studies have been performed at 3 Tesla with the resulting benefit of increased signal-to-noise ratio [21–23]. The technique has been validated *in vitro* against constant flow but to date, there has been no validation *in vivo* where the flow is pulsatile and the vessels are highly mobile. Likewise, while the inter-study reproducibility of CMR measured parameters of coronary blood flow have been assessed, they have not been directly compared against Doppler values.

Measurement of absolute coronary flow at any time point requires accurate assessment of the mean flow velocity and the cross-sectional area, both of which require high spatial resolution. Assessment of the temporal patterns of coronary blood flow through the cardiac cycle, on the other hand, requires high temporal resolution to resolve temporal detail. For such purposes, spatial resolution can be traded for high temporal resolution although this leads to increased partial volume averaging and reduced velocities at any time point. The aim of this study is to develop a spiral phase velocity mapping technique with sufficiently high temporal resolution to allow assessment of the temporal pattern of coronary artery blood flow in a breath-hold and to directly compare the flow patterns obtained with those from gold-standard Doppler guide wire studies performed in humans. The inter breath-hold reproducibility of CMR assessment of blood flow patterns is also assessed.

## Methods

The study was approved by a National research ethics committee and all subjects gave written informed consent.

### Doppler guide wire study

Coronary haemodynamics at rest were studied at the time of clinically indicated coronary angiography in 18 patients (12 male, mean (+/−SD) age = 56 (+/−13) years, range 33 – 73 years). The clinical indication was typically shortness of breath and/or chest pain. Fourteen patients had hypertrophic cardiomyopathy (HCM), 1 had syndrome X and 3 had atypical chest pain with risk factors for coronary artery disease. Of these 18 patients, 56 % had smooth angiographically normal coronary arteries and 44 % had minor irregularities (30 % of luminal diameter or less) but no flow limiting stenoses. Angiography was performed using radial access in 50 % of patients and using femoral access in the remainder. In 4 of the radial access patients, 300mcg of nitrates were given following insertion of the radial sheath. No nitrates were given if there was any likelihood of outflow tract obstruction. Following completion of diagnostic coronary angiography, heparin was administered and pressure and flow velocity measurements were made using an intra-arterial pressure and Doppler velocity wire (Combowire, Volcano Therapeutic) positioned in the proximal left anterior descending (LAD) artery and in the proximal right coronary artery (RCA). Resting measurements were taken approximately 20 min after administration of any intra-arterial nitrate when blood pressure and heart rate had returned to resting levels. A fluoroscopic recording was made at each location to document the location of the measurements and to act as a reference for the subsequent CMR study. Flow velocity and electrocardiogram measurements were taken at 5 ms intervals for a period of 60 s during quiet free-breathing using a National Instruments multifunction I/O card (DAQCard-6062E) and a customised acquisition layer based upon a Labview software shell. The detected peak velocity within the Doppler sample volume was overlaid on the Doppler traces. In vessels which showed periods of systolic flow reversal, bi-directional peak detection was attempted although this generally resulted in a noisier peak velocity trace. If too noisy, standard uni-directional peak velocity tracking was used instead and the data were analysed as discussed in the next section. A text file was output containing the peak velocity within the sample volume at 5 ms intervals. The timing of the QRS complex of the ECG was also output.

### CMR study

An interleaved spiral phase velocity sequence was developed on a 3 Tesla Magnetom Skyra MR scanner (Siemens AG Healthcare Sector, Germany) equipped with an 18-element cardiac coil and a 48-element spine coil. Spatial resolution was traded to give high temporal resolution within a comfortable breath-hold period so that fine details in the temporal flow pattern could be visualised for comparison with the Doppler guidewire. At the same time, spiral readout duration was limited to < 12 ms to minimise problems of off-resonance blurring. A 1–1 water excitation (duration = 3 ms) was implemented which eliminated off-resonance blurring of fat and full k-space coverage was achieved in 8 spiral interleaves of 11.75 ms duration. Phase map subtraction of datasets with symmetric bi-polar velocity encoding gradients resulted in through-plane velocity maps where a phase shift of +/−180^0^ represented a flow velocity of +/−30 cm/s. Following a single dummy cycle, these velocity encoded datasets were acquired in alternating cardiac cycles in an end-expiratory breath-hold of 17 cardiac cycles duration. The sequence TE was 5.2 ms, and the TR was 19 ms. Data were reconstructed online following gridding onto a 256 × 256 matrix using a standard gridding algorithm [24]. The number of coil elements used was limited to 6 from the anteriorly-positioned cardiac coil and 6 from the posterior spine coil in order to reduce reconstruction time and to minimise wrap. The slice thickness was 8 mm, the spatial resolution 1.4 × 1.4 mm (reconstructed to 0.7 × 0.7 mm through zero-filling) and the repeat time (acquired temporal resolution) 19 ms. Retrospective ECG gating allowed full coverage of the entire cardiac cycle in 50 cine frames, the reconstructed temporal resolution depending on the subjects’ heart rates.

Multiple early diastolic breath-hold transverse segmented gradient echo scout acquisitions (TE/TR: 3.3 ms/7 ms, acquired resolution: 1 mm × 1 mm × 4 mm, acquisition window 110 ms) were acquired in each subject to ascertain the path of the coronary artery of interest. From these, oblique and double oblique images were acquired showing the path of the artery in-plane, followed by a through-plane acquisition in a straight section of the proximal artery, matched as closely as possible to the location of the invasive measurement. Spiral coronary artery phase velocity maps were then acquired in the same location. Sensitivity to off-resonance was minimised by localised second-order shimming and frequency adjustment based on the signal from a user-defined region of interest positioned over the heart. For right coronary studies, an additional breath-hold spiral phase velocity mapping acquisition was performed using fat-excitation [20]. This was used to correct the data for the through-plane velocity of the vessel, as discussed in the analysis section. In 15 vessels (10 LAD and 5 RCA), the spiral acquisitions were repeated to allow an analysis of inter breath-hold reproducibility of the temporal flow patterns.

While ideally the CMR study would be carried out the same day as the invasive study, this was not always practicable. Blood pressure during the CMR study was measured using a brachial cuff.

### Analysis – Doppler guidewire study

A composite Doppler velocity time-curve was assimilated for each vessel as the average of 10–20 cardiac cycles of data. If the bi-directional peak flow detection algorithm failed to work reliably - which was most often the case - the unidirectional peak flow detection algorithm was used instead. The Doppler traces often showed a degree of mirror artefact whereby the trace is reflected about the horizontal zero velocity line. In subjects with reverse systolic flow, the unidirectional peak flow detection picked up the mirrored (positive) velocity rather than the negative velocity. If it was clear from the relative intensities of the real and mirrored signals that the flow was indeed negative, then that peak flow velocity was reversed to record a negative value. If it was not clear from the traces whether the flow was positive or negative - usually when the absolute velocity was very low or, less often, when the mirror intensity was low - then that section of the trace (typically 50–100 ms) was eliminated (ie treated as missing data) for the purposes of comparison with CMR.

### Analysis – CMR study

Due to partial volume averaging within the relatively large pixels (1.4 mm × 1.4 mm, reconstructed to 0.7 mm × 0.7 mm), the peak pixel velocities in the CMR study would be considerably less than the corresponding Doppler guidewire peak velocities. Single pixel values are also noisy. Consequently, rather than determining peak pixel velocities, the CMR velocities determined are a mean over the cross-sectional area of the vessel - for parabolic flow, these mean velocities would be expected to be ~50 % of the peak velocities. For the CMR data in the subset of 15 subjects with repeat breath-hold acquisitions, velocity-time curves were generated for each breath-hold by two independent observers using semi-automatic custom MATLAB software. This requires the user to mark the centre of the vessel on the cross-sectional segmented gradient echo scout image and following multi-level thresh-holding [25], a circular ROI is automatically defined around the coronary artery using a modified Hough transform based algorithm [26]. This initial ROI is copied to the first frame of the spiral magnitude dataset which is put through a spatial band pass filter to identify objects of similar size. A search for local maxima within a specified range of the initial ROI center locates these objects and the one closest to the initial position is selected as the location of the artery in that time-frame. This new ROI location is copied to the corresponding velocity map and to the next frame in the spiral magnitude dataset where the process is repeated, thereby automatically tracking the artery from frame to frame of the acquisition – the ROI size remains fixed throughout the cardiac cycle. The resulting velocity-time curve is a composite of the through-plane velocity of coronary blood flow and the through-plane velocity of the vessel itself. For the LAD, the velocity of a tracked region of nearby myocardium is used as a marker of the through-plane velocity of the vessel [13]. For the RCA, the adjacent myocardium is too thin to use as a correction and the velocity of a tracked region of interest in the surrounding epicardial fat, as seen on the fat-excitation breath-hold acquisition, is used instead [20]. For these 15 vessels, the velocity-time curves output by the semi-automatic analyses were validated against those derived following manual definition and tracking of the coronary ROIs.

The following parameters were extracted from each manually and semi-automatically derived velocity-time curve: peak systolic velocity (PSV), peak diastolic velocity (PDV), time to peak systolic velocity (TPSV), time to peak diastolic velocity (TPDV) and mean velocity through the cardiac cycle (MV). For each vessel, flow was calculated as the average velocity through the cardiac cycle multiplied by the cross-sectional area. In a subset of 15 vessels (10 LAD and 5 RCA), the results of semi-automatic analyses were compared with manual analyses using the intraclass correlation coefficient and Bland Altman analysis [27]. The inter-observer variability and the inter breath-hold reproducibility of the semi-automatic analysis were determined in the same way.

Following this assessment of the semi-automatic technique, all data comparisons with Doppler were performed using the results of the semi-automatic analyses.

### Comparison with Doppler

From each Doppler and CMR velocity time curve, peak systolic velocity (PSV), peak diastolic velocity (PDV), and mean velocity through the cardiac cycle (MV) were determined. For each parameter, CMR data were plotted against Doppler data and simple linear regression performed. Coronary blood flow velocity increases with heart rate [28, 29] so to correct for physiological differences in heart rate between the two acquisitions, these analyses were repeated after normalising the CMR data to the same heart rate as the Doppler data. To assess temporal flow patterns, the ratios of the peak diastolic velocities to the peak systolic velocities (PDV/PSV) were compared between techniques. In addition, after shifting the CMR velocity-time curves to account for small changes in the ECG triggering between the CMR and Doppler studies, the CMR velocities throughout the cardiac cycle were plotted against the corresponding Doppler velocities for each vessel. The relationship between them was assessed with simple linear regression analysis and the coefficient of determination (R^2^).

All analyses were performed using the IBM SPSS Statistics 19 Package and a *p* value <0.05 was regarded as statistically significant.

## Results

Good quality Doppler data were available in 18 LAD arteries and in 9 RCAs. Good quality CMR data were acquired in 23 (85 %) of these vessels (15 LAD arteries and 8 RCA). Of the poor quality CMR studies, the 3 LAD studies showed off-resonance blurring while the RCA study was inadvertently acquired at the approximate origin of a branch vessel with a branch angle close to 90^0^ which would be expected to disturb the flow pattern ([30]). The mean cross-sectional area was 12.5 +/−3 mm^2^ (or 25.5 +/−6.7 reconstructed pixels), as measured by the semi-automatic technique. While overall, there was no significant difference in heart rate between the non-invasive and invasive studies (66.6 +/−12.2 *vs* 63.5 +/−11.4 beats per minute, *p* = 0.14), the standard deviation of the paired differences between the two studies was high (9.6 beats per minute) and the heart rate at the time of the CMR study ranged from 13 beats per minute lower than in the corresponding Doppler study to 21 beats per minute higher. The central systolic and diastolic blood pressures measured during the invasive Doppler study were significantly higher than the brachial cuff pressures measured during the CMR study (137 +/−21 *versus* 115 +/−18 mmHg and 81 +/−13 *versus* 67 +/−10 mmHg, both *p* < .001 respectively).

**Fig. 1:**
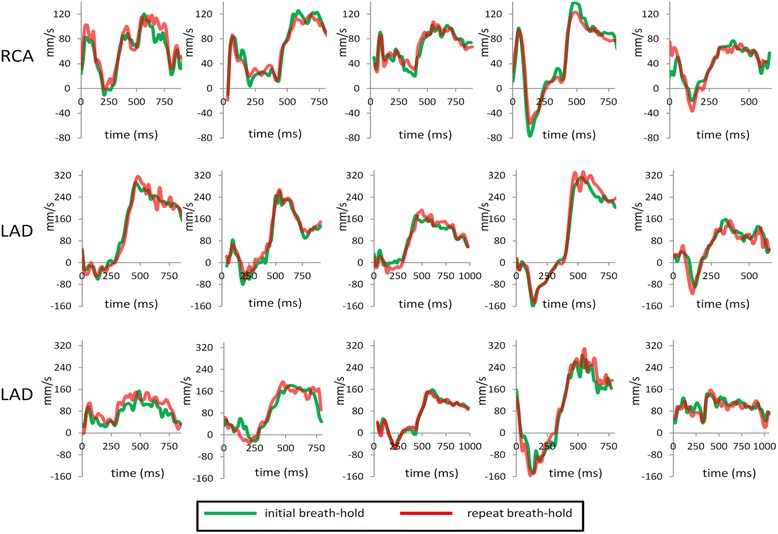
CMR velocity-time curves assessed with the semi-automatic technique in repeated breath-holds (*red and green*) in 5 right coronary arteries (*top*) and 10 left anterior descending arteries (*middle and bottom*)

The semi-automatic analysis technique enabled velocity-time curves of coronary blood flow velocity to be produced in typically < 5 min with minimal user interaction. This included time to review the coronary and through-plane correction ROIs on all 50 cine frames (magnitude images and velocity maps). Manual analysis took approximately 30 min per vessel. Table 1 shows the results of manual and semi-automatic analyses of MV, flow, PSV, TPSV, PDV and TPDV in 15 vessels, together with the inter-observer reproducibility of these parameters for the semi-automatic analyses. Table 2 shows the inter breath-hold reproducibility for each of two observers. Figure 1 shows the inter breath-hold reproducibility of the temporal flow patterns in the 5 RCA and in the 10 LAD arteries, as measured with the semi-automatic technique. While the shape of the plots varies from patient to patient and includes a number of vessels with reverse flow during systole, in each case, the temporal flow patterns from one breath-hold to the next are highly similar.

**Table 1:** Comparison between semi-automatic and manual analyses of coronary blood flow parameters (mean +/−SD of paired differences, intraclass correlation coefficient (ICC)) in 15 vessels, together with inter-observer reproducibility (semi-automatic method) of the same variables

	MANUAL *VERSUS* SEMI-AUTOMATIC ANALYSIS			INTER-OBSERVER REPRODUCIBILITY (SEMI-AUTOMATIC ANALYSIS)		
	mean +/−SD	mean (+/−SD) differences	ICC	mean +/−SD	mean (+/−SD) differences	ICC
mean velocity (mm/s)	78.7+/−25.8	−0.9+/−9.8	0.93	81.0+/−23.9	−3.7+/−7.3	0.95
flow (ml/min)	54.9+/−23.7	−10.5+/−11.0*	0.81	61.4+/−26.3	−2.7+/−5.7	0.98
PSV (mm/s)	72.5+/−35.2	−1.7+/−17.0	0.98	74.5+/−33.1	−1.4+/−16.0	0.98
TPSV (ms)	115.3+/−57.3	5.5+/−19.4	0.94	114.1+/−55.6	−1.9+/−13.3	0.97
PDV (mm/s)	171.0+/−75.4	−1.3+/−24.7	0.95	173.1+/−69.9	−3.3+/−16.2	0.98
TPDV (ms)	515.2+/−95.1	−3.0+/−50.2	0.90	521.4+/−106.7	−9.4+/−28.5	0.96

The means of the manual and semi-automatic values (+/−SD) and of the two observers values are included for reference. The ICC is calculated for absolute agreement (single measure). *MV* mean velocity, *PSV* peak systolic velocity, *TPSV* time to peak systolic velocity, *PDV* peak diastolic velocity and *TPDV* time to peak diastolic velocity. (* *p* < .001)

**Table 2:** Inter breath-hold comparison between semi-automatic analyses of coronary blood flow parameters (mean +/−SD of paired differences, intraclass correlation coefficient (ICC)) in 15 vessels for two observers (obs 1 and obs2)

	mean+/−SD		mean (+/−SD) differences		ICC	
	obs1	obs2	obs1	obs2	obs1	obs2
mean velocity (mm/s)	80.4+/−23.9	82.0+/−25.7	−2.4+/−6.9	1.7+/−7.4	0.96	0.96
flow (ml/min)	61.1+/−26.7	62.4+/−28.0	−1.9+/−4.8	0.8+/−5.5	0.99	0.96
PSV (mm/s)	73.4+/−33.3	74.7+/−31.1	1.7+/−15.8	−0.1+/−12.6	0.98	0.99
TPSV (ms)	119.0+/−53.3	113.0+/−59.2	−4.4+/−22.1	−2.7+/−20.1	0.93	0.95
PDV (mm/s)	173.4+/−72.5	173.2+/−74.4	−3.5+/−11.6	2.9+/−21.4	0.99	0.97
TPDV (ms)	516.8+/−90.8	536.1+/−103.4	−0.1+/−49.3	−20+/−70.3	0.95	0.90

The means of the two breath-hold values (+/−SD) are included for reference for each observer. The ICC is calculated for absolute agreement (single measure). *MV* mean velocity, *PSV* peak systolic velocity, *TPSV* time to peak systolic velocity, *PDV* diastolic peak velocity and *TPDV* time to diastolic peak velocity

**Fig. 2:**
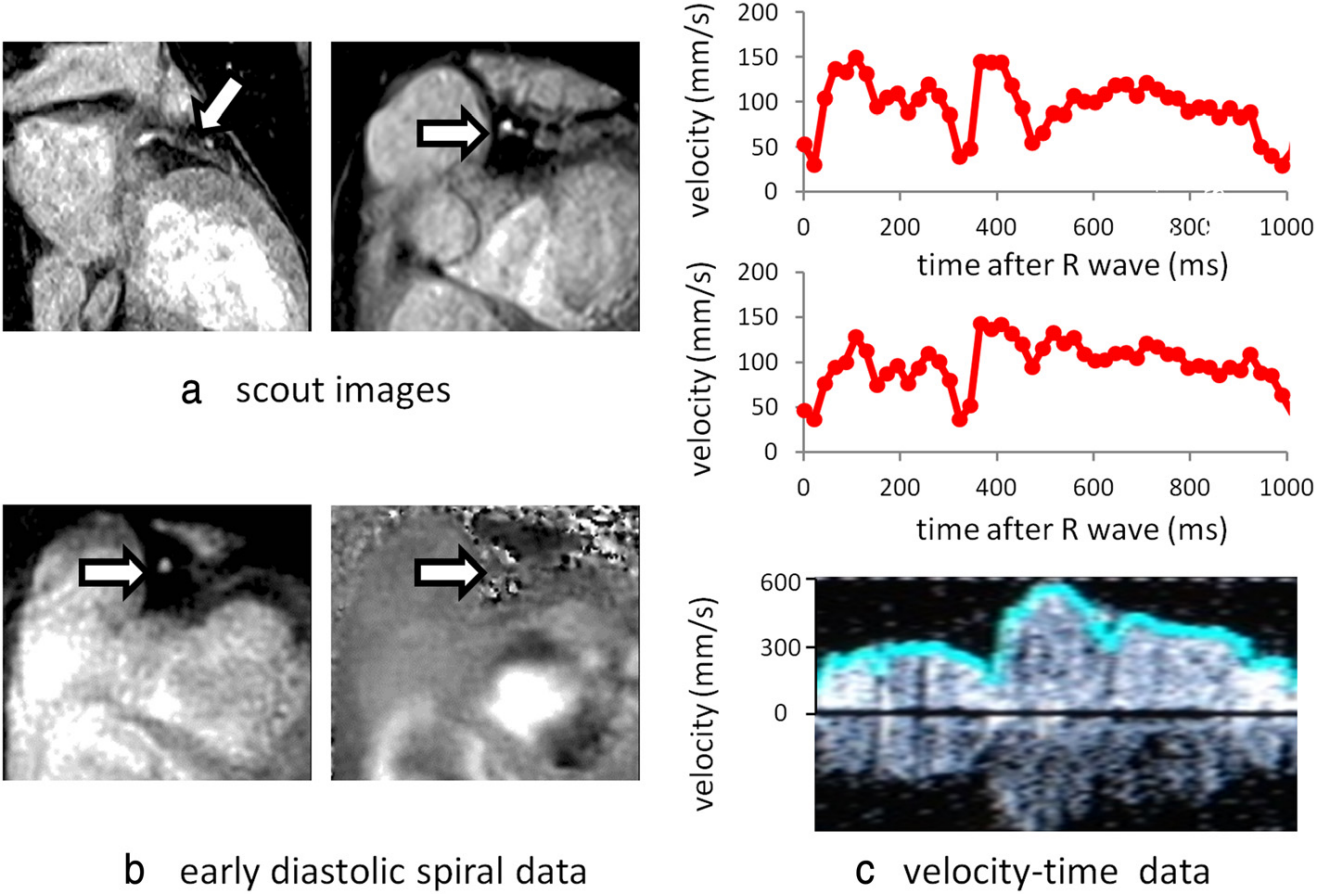
**a** Segmented gradient echo scout images showing in-plane (*left*) and proximal through-plane (*right*) left anterior descending coronary artery (*arrows*). **b** Single early diastolic frame from the corresponding high temporal resolution spiral phase velocity mapping study acquired with water-excitation (*WE*) (*magnitude image on left, velocity map on right*). **c** CMR velocity-time curve before (*top*) and after (*middle*) correction for through-plane velocity of the vessel and corresponding Doppler guide wire trace (*bottom*). On the Doppler guidewire trace, the peak pixel velocity within the sample volume is highlighted in blue

**Fig. 3:**
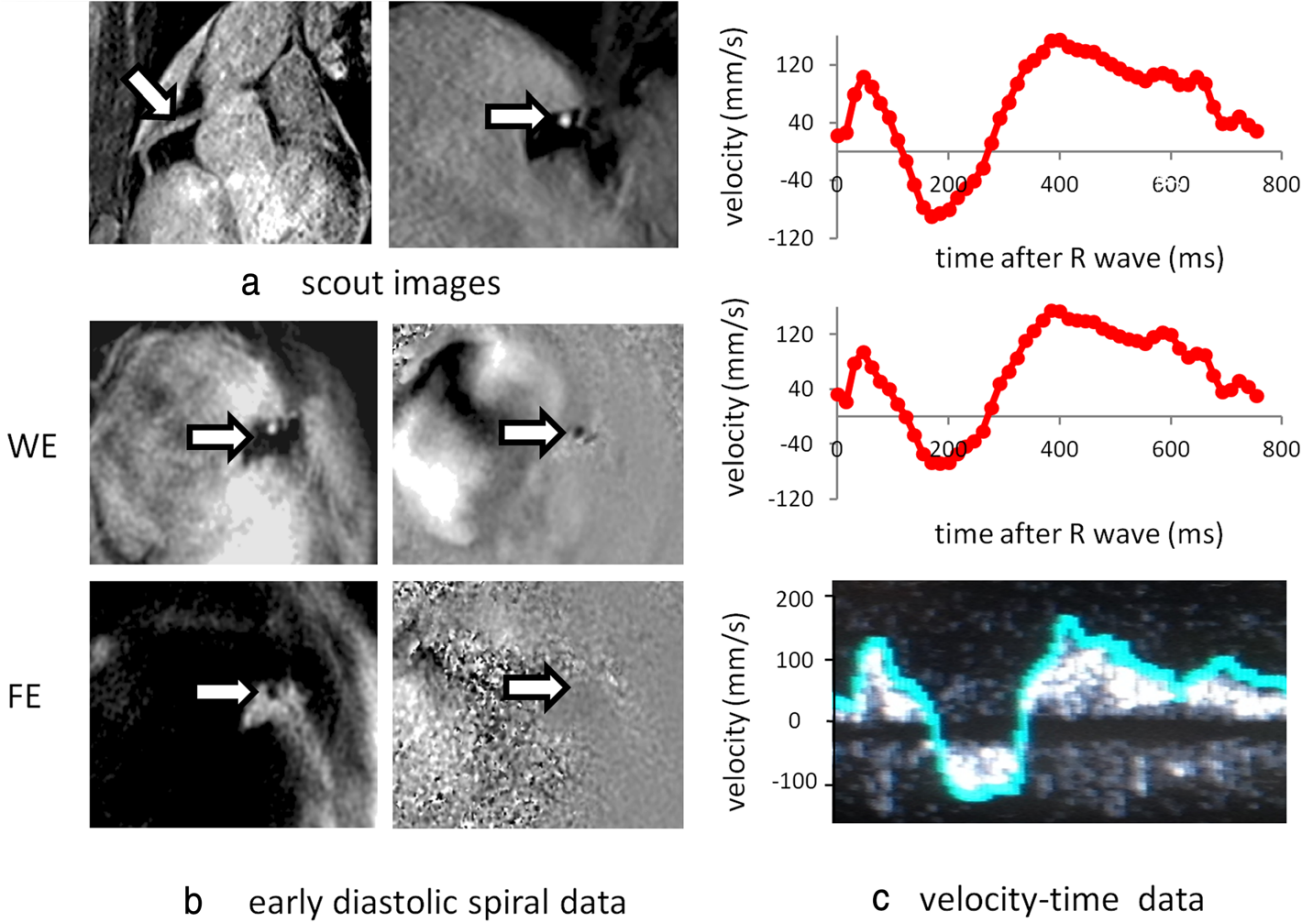
**a** Segmented gradient echo scout images showing in-plane (*left*) and proximal through-plane (*right*) right coronary artery (*arrows*). **b** Single early diastolic frame from the corresponding high temporal resolution spiral phase velocity mapping study acquired with water-excitation (*WE*) (*magnitude image on left, velocity map on right*) together with corresponding fat-excitation (*FE*) images. **c** CMR velocity-time curve before (*top*) and after (*middle*) correction for through-plane velocity of the vessel and corresponding Doppler guide wire trace (*bottom*). On the Doppler guidewire trace, the peak pixel velocity within the sample volume is highlighted in blue

**Fig. 4:**
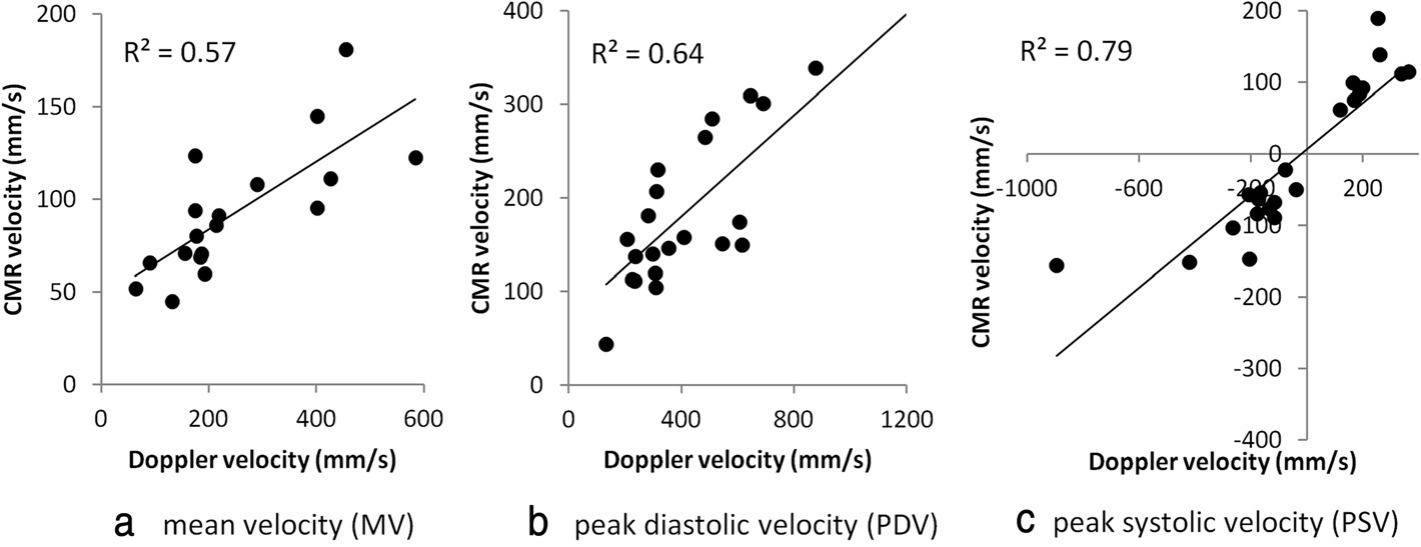
CMR measurement of mean velocity (*MV*) (**a**), peak diastolic velocity (*PDV*) (**b**) and peak systolic velocity (*PSV*) (**c**) against Doppler values after scaling to same RR interval in 23 vessels

Example LAD and RCA studies analysed with the semi-automatic technique are shown in Figs. 2 and 3 respectively. Figure 4 shows plots of CMR measured mean velocity (a), PDV (b) and PSV (c) against Doppler measured values, after normalising the CMR data to the same heart rate as the corresponding Doppler data. The negative PSV values in (c) reflect early systolic reverse coronary flow which is an expected feature in patients with HCM [31]. There is an approximately linear relationship between the two techniques for all three parameters with coefficients of determination ranging from moderate for MV (*R*^2^ = 0.57) to good for PDV (*R*^2^ = 0.64) to very good for PSV (*R*^2^ = 0.79). The heart rate normalised CMR velocities are typically ~40 % of the Doppler values (MV: 93 +/−35 mm/s vs 251 +/−143 mm/s, *p* < .0001, PDV: 199 +/−94 mm/s vs 468 +/−275 mm/s, *p* < .0001; PSV: 95 +/−41 mm/s vs 229 +/−174 mm/s, *p* < .0001).

**Fig. 5:**
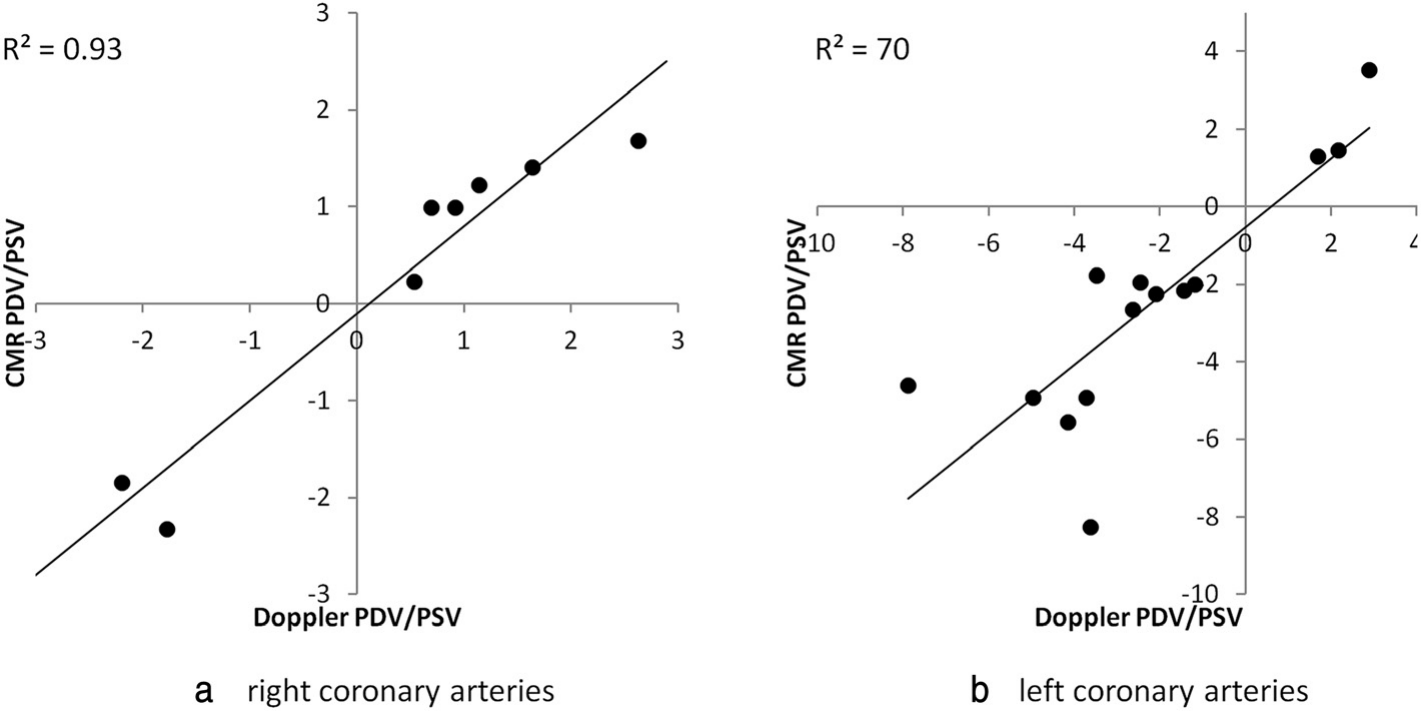
CMR measurement of the ratio of peak diastolic velocity to peak systolic velocity (*PDV/PSV*) plotted against Doppler values in 8 right coronary arteries (**a**) and 14 left anterior descending arteries (**b**). (*One LAD artery was excluded as it had no systolic peak*)

Figure 5 show plots of CMR *versus* Doppler values of PDV/PSV for the RCAs (a) and LAD vessels (b). The relationships are linear with slopes close to unity (RCA: slope 0.90, LAD: slope 0.89) with high coefficients of determination (0.93 and 0.70 for the right and left arteries respectively).

**Fig. 6:**
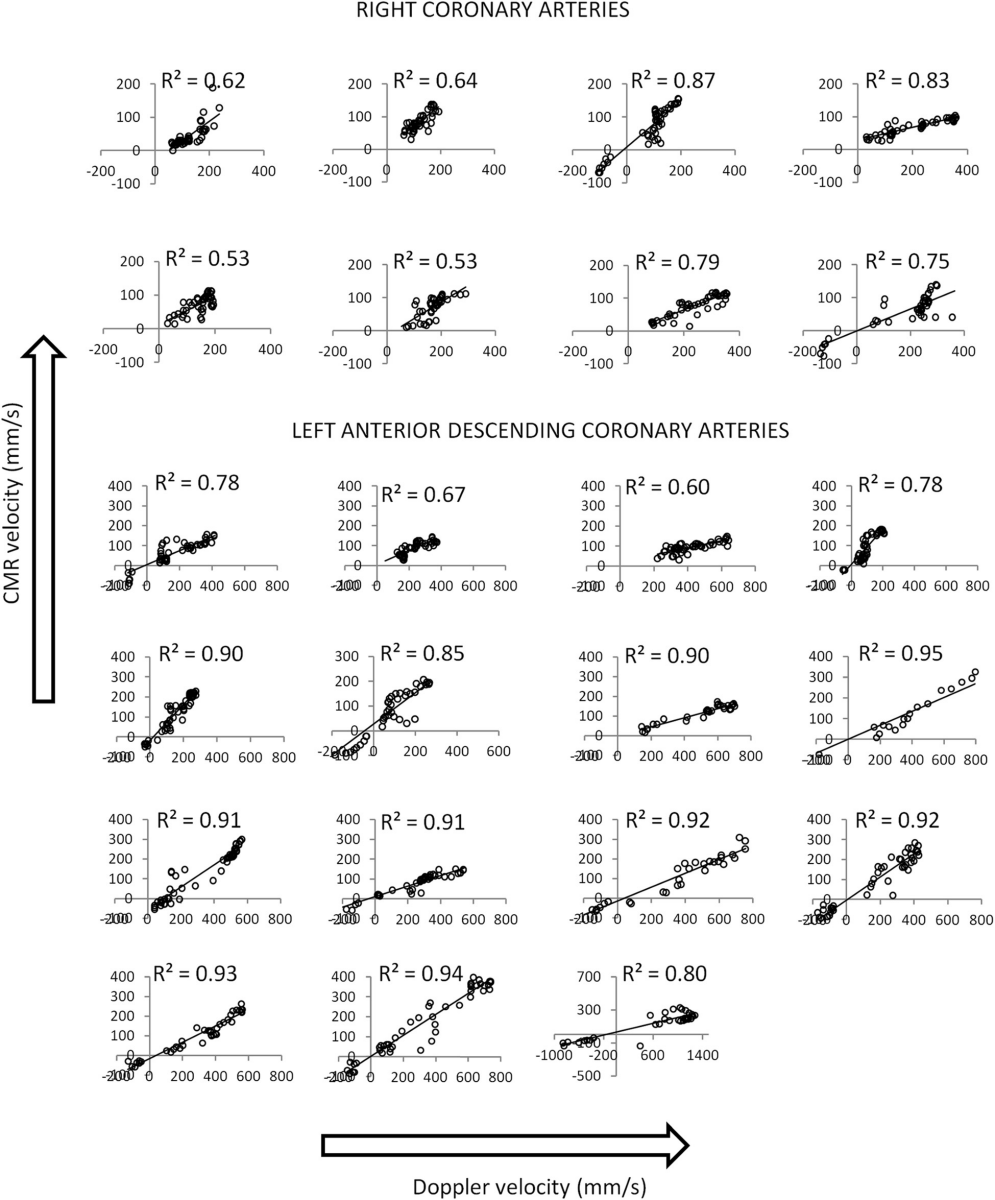
CMR velocity at all points in the cardiac cycle against Doppler velocity for all 8 right coronary arteries and all 15 left anterior descending arteries. Linear regression lines are superimposed and the R^2^ values presented for each. (For all plots, x-axis is Doppler velocity in mm/s; y-axis is CMR velocity in mm/s)

**Fig. 7:**
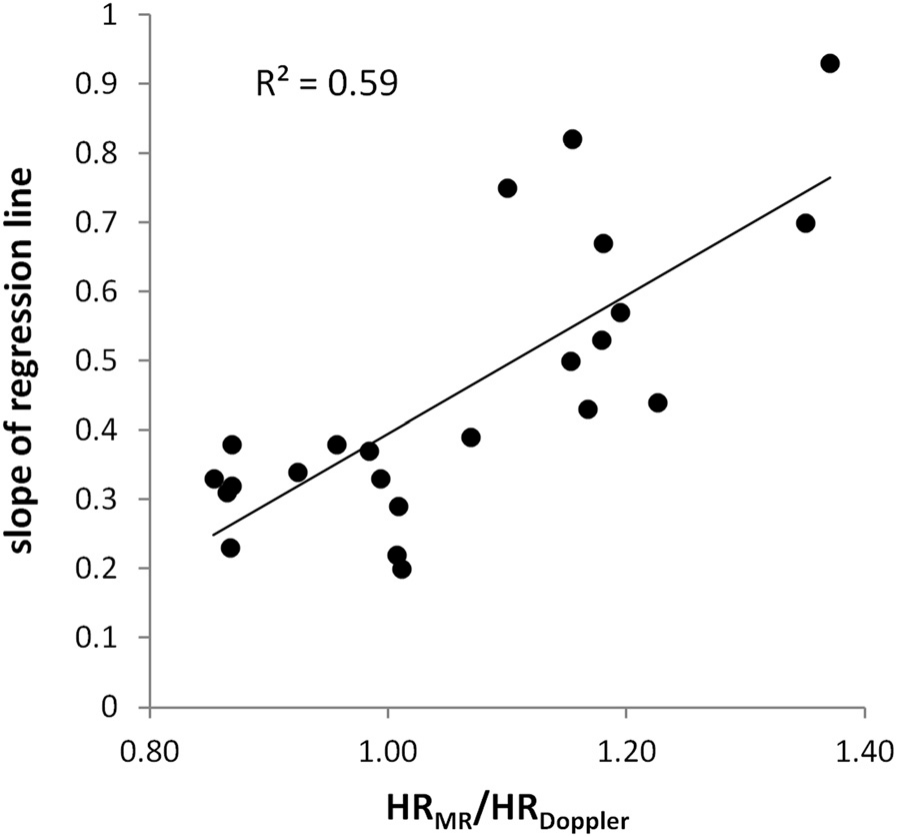
Slope of CMR velocity *versus* Doppler velocity regression lines of Fig. 6 against the ratio of the heart rate during the CMR study (*HR*_*MR*_) to that in the Doppler study (*HR*_*Doppler*_)

Figure 6 shows plots of the CMR measured velocities at all time points in the cardiac cycle against the corresponding Doppler velocities for all 23 vessels. Linear regressions are superimposed on each plot together with the coefficient of determination. The slopes of the regression plots vary from vessel to vessel (mean (+/−SD): 0.45 +/−0.20, range 0.20–0.93) which results in the scatter in MV, PSV and PDV seen in Fig. 4. Higher regression slopes are seen in patients in whom the heart rate during the CMR study was higher than that during the invasive study, as shown in Fig. 7. In studies where the heart rate in the CMR study was within 15 % of that in the invasive study, the regression slope was 0.35 +/−0.13. However, regardless of the value of the slope, the relationships between the CMR and Doppler velocities are linear with high coefficients of determination (mean R^2^ (+/−SD) = 0.79 +/−0.13). All *R*^2^ values were > 0.5 and in 74 % of vessels, *R*^2^ was ≥ 0.75.

## Discussion

We have developed a high temporal resolution spiral phase velocity mapping sequence which allows the acquisition of temporal patterns of coronary artery blood flow in the left and right coronary arteries. The implementation of retrospective ECG gating allows acquisition throughout the entire cardiac cycle for more accurate determination of MV. Custom MATLAB software has enabled rapid analysis of the velocity maps, generating through-plane vessel-motion corrected data in typically < 5 min with minimal user interaction. We have shown that this software produces results in good agreement with considerably more laborious manual analysis for MV, PSV, TPSV, DPV and TPDV. For flow, however, there is a significant difference between the manual and semi-automatic techniques due to a significant difference in measured cross-sectional areas (12.5 +/−3.3 mm^2^*versus* 10.9 +/−3.2 mm^2^, *p* = 0.03). This corresponds to a mean vessel radius of 1.9 mm when assessed with the manual technique and 2.0 mm when assessed with the semi-automatic technique, the difference being very small relative to the pixel size. The semi-automatic technique shows high inter-observer reproducibility for all parameters. The inter breath-hold reproducibility of the technique is excellent for MV, flow, PSV, TPSV and DPV. The slightly less good inter breath-hold reproducibility for TPDV reflects the fact that this peak is broader and therefore more difficult to locate.

We have directly compared CMR measures of MV, PDV and PSV with those from invasive Doppler studies and found moderate – good correlation (*R*^2^ = 0.57–0.79) between the two after normalisation to the same RR-interval. The temporal flow patterns have been assessed by comparing the diastolic to systolic velocity ratios and by performing linear regression analysis of CMR velocities *versus* Doppler velocities in each individual vessel. The agreement between CMR and Doppler assessed PDV/PSV is excellent (R^2^ values of 0.93 and 0.71 for left and right arteries respectively) with a slope close to unity (0.89 for the LAD arteries, 0.90 for the RCAs). Regression plots of the CMR measured velocities against the Doppler measured velocities throughout the cardiac cycle show good linear correlation for all vessels (all *R*^2^ > 0.5; 74 % of vessels *R*^2^ ≥ 0.75).

In our study, good quality CMR images were obtained in 23 of the 27 vessels studied (85 %). Reducing the spiral readout duration may result in a higher number of good quality images (and improved temporal resolution) through reduced off-resonance blurring, but this would impact on the spatial resolution and/or the breath-hold duration achievable*.*

There have been a few previous reports using spiral phase velocity mapping in the coronary arteries [18–20], including a recent one at 3 Tesla [21]. However, our study is the first to directly compare the results of spiral phase velocity mapping with those from an invasive gold-standard Doppler guidewire study. In addition, it incorporates retrospective ECG gating to cover the entire cardiac cycle and has a higher temporal resolution (19 ms compared to 30 ms [19] or 33 ms [21]) although this is at the expense of decreased spatial resolution (1.4 mm × 1.4 mm (reconstructed to 0.7 mm × 0.7 mm) compared to 0.8 mm × 0.8 mm (reconstructed to 0.7 mm × 0.7 mm) [21]. The spiral readout duration in the current study is lower (11.8 ms *versus* 26 ms) which reduces off-resonance blurring and the breath-hold duration is also reduced (17 cardiac cycles compared to 24 cardiac cycles) [21]. While we have reported inter breath-hold reproducibility of flow parameters, inter-study reproducibility (which includes patient repositioning) has been reported by Brandt et al. [21] (mean difference +/−SD paired differences: PSV−6 +/−32 mm/s, PDV−12 +/−20 mm/s). We would expect the inter breath-hold reproducibility to be better than the inter-study reproducibility as it doesn’t include the effects of patient repositioning. While our results are consistent with this (inter breath-hold reproducibility of PSV 1.7 +/−15.8 mm/s, and of PDV 3.5 +/−11.6 mm/s), it is difficult to compare the results of the two studies directly as in the previously published study, the PSV and PDV values were determined as the peak pixel value, rather than the mean over the cross-sectional area. They were also obtained in RCAs only and in a population of very young, healthy volunteers rather than in patients being investigated for chest pain and shortness of breath.

For the CMR velocity-time curve analysis, the coronary artery ROI is automatically defined on the diastolic segmented gradient echo scout image, rather than on the spiral magnitude data, as this has higher spatial resolution (1 mm × 1 mm rather than 1.4 mm × 1.4 mm) and is less susceptible to off-resonance blurring. The analysis method developed assumes that the cross-section of the coronary artery is circular which, provided that the imaging plane has been carefully set-up on a non-diseased section of artery, is reasonable. X-ray fluoroscopy studies have shown that the coronary artery area changes by ~10 % through the cardiac cycle [32]. However, the spatial resolution of our acquisition technique was traded for high temporal resolution and is not sufficient to pick this up accurately. We have consequently used a fixed region of interest size throughout the cardiac cycle.

The CMR measured velocities at any time point in the cardiac cycle are typically ~40 % of the corresponding Doppler velocities. This is to be expected as CMR is measuring the mean velocity within a ROI encompassing the coronary artery while with Doppler, the recorded velocities are the peak values within the small sample volume positioned in the flow center line. This result is in line with a previous study showing that CMR measured PDV using a segmented gradient echo technique (navigator-gated with an acquisition window of 45 ms) was 37 % of the corresponding Doppler value [15]. Comparing Doppler values to the peak pixel velocities within the CMR ROIs, rather than the mean values, would increase this percentage but the relatively low spatial resolution of the CMR acquisition (1.4 × 1.4 mm, reconstructed to 0.7 × 0.7 mm) would still result in spatial averaging of the velocity profile across the pixel and the result would depend on the exact location of that pixel relative to the flow center line. The CMR data would therefore continue to underestimate the Doppler values and at the same time, as the CMR data from a single pixel would be noisier than that from the cross-sectional average, the correlation between the two would be less good. Ultimately, we are interested in the temporal patterns of blood flow through the cardiac cycle rather than the absolute agreement with Doppler and consequently, we used the mean value. For assessment of absolute coronary flow, a potential alternative approach would be Fourier velocity encoding which, with multiple velocity encodings, may give more accurate measures through reduced partial volume effects. However, this would require some form of respiratory gating which would result in long and unpredictable scan times which would be a particular problem for future studies carried out under pharmacological stress.

The invasively measured systolic and diastolic blood pressures at the time of the invasive study were significantly higher than those measured non-invasively at the time of the CMR study. These differences may reflect differences in the measurement techniques or in the physiological state of the patients or a combination of both. In addition, while overall there was no significant difference between the heart rates in the two studies, on an individual patient basis, the paired heart rate differences ranged from −21 to +13 beats per minute. Such changes in blood pressure and heart rate will change the coronary blood flow velocity (MV, PDV and PSV) and are in part responsible for the scatter in the plots of Fig. 4. While we had originally planned to perform the invasive and non-invasive studies on the same day to limit these differences, this was not always practical (one invasive site was 350 km from the main study site) and the majority of patients preferred them to be performed on different days. Previous studies [28, 29] have shown that the change in blood flow velocity with increasing heart rate is approximately linear within the range of physiological heart rates and a simple scaling of the CMR measured velocities to the same heart rate as the corresponding Doppler velocities resulted in the plots shown in Fig. 4. This normalisation assumes a 1:1 relationship between flow velocity and heart rate and that all time points in the cardiac cycle are equally affected. This seems reasonable given that the ratio PDV/PSV between CMR and Doppler studies is close to unity regardless of differences in heart rate between the studies ie the absolute values of velocity change with heart rate but the shape of the temporal flow profile is fixed. Further evidence that the shapes of the flow profiles are the same, regardless of heart rate or pressure, is presented in Fig. 6 where regression of CMR and Doppler data through the cardiac cycle are plotted for each individual vessel. In all cases, a linear correlation is seen (all *R*^2^ > 0.5) although as expected from Fig. 4, the slopes of the regression analyses in the individual vessels are highly variable. The linear relationships between invasive and non-invasive velocities are strong with the mean R^2^ being 0.79 with a small standard deviation (0.13) and with 74 % of vessels having an *R*^2^ value >0.75. In this study, there has been no attempt to account for differences in pressure between the two studies. The rationale for this is twofold: (a) although there is a correlation between invasively and non-invasively determined pressures, there are significant differences between them [33] and (b) it is expected that autoregulation maintains coronary blood flow over a limited range of pressure [34] and it is therefore debatable whether such a correction is necessary.

The invasive study results in Doppler traces showing the velocities present within the sample volume through the cardiac cycle as a function of time with the peak velocity at any time-point highlighted to show the temporal pattern of flow. It is this peak velocity that is used to generate velocity-time curves for comparison with the CMR data. Interference between the positive and negative channels of the signal receiver and the lack of a filter on the beam former often results in a mirror artefact in the Doppler traces whereby the trace is reflected about the zero velocity line, as in Fig. 2. In cases of positive flow velocity, the uni-directional peak velocity detection software always detects the main (un-mirrored) signal. However, in cases of reverse flow – which is a common finding in patients with HCM – it will detect the mirrored signal. In this study, if it was clear from the relative intensities of the real and mirrored signals that the flow velocity was indeed negative, then that peak flow velocity was reversed to record a negative value. If it was not clear from the traces whether the flow was positive or negative – usually when the absolute velocity was very low - then that section of the trace (typically 50–100 ms) was eliminated for the purposes of comparison with CMR. This complication could have been avoided by studying healthy volunteers where flow velocity is always positive, but acquiring invasive Doppler data in healthy volunteers in the absence of clinical indications is unethical. Alternatively, in high SNR cases of reverse flow where mirroring was not a problem, a bi-directional peak velocity detection algorithm could be used (Fig. 3).

While the Doppler guidewire is commonly regarded as the gold-standard for coronary artery blood flow velocity assessment, in our study population, it was more difficult to obtain good quality studies in the proximal RCA (9 vessels) than in the LAD artery (18 vessels), despite having highly experienced operators. This was potentially due to the higher mobility of the RCA which was exacerbated in our study where 14 of the 18 patients had HCM with hyper-contractility of the left ventricle. Consequently, the number of right and left arteries in our study was imbalanced. A further practical difficulty was ensuring that CMR and Doppler studies were carried out in the same location in the vessel of interest. This was minimised by access to a fluoroscopic image from the invasive study at the time of the CMR study, allowing the location of the CMR measurement to match the Doppler location as best as possible while ensuring the slice location was away from branch points and in a straight section of the vessel. However, SNR constraints required that the CMR slice thickness was relatively large (8 mm) and the flexibility of slice positioning was consequently limited.

## Conclusions

We have developed a high temporal resolution spiral phase velocity mapping technique for the assessment of coronary artery blood flow at 3 Tesla. While, as expected, absolute measures of flow parameters are underestimated due to partial volume averaging, the temporal patterns of coronary blood flow velocity, are highly similar to those obtained in invasive Doppler guidewire studies. We conclude that this technique may be used to assess temporal flow patterns non-invasively which provides important information on disease state.

## Abbreviations

TR: Repeat time
TE: Echo time
PSV: Peak systolic velocity
TPSV: Time to peak systolic velocity
PDV: Peak diastolic velocity
TPDV: Time to peak diastolic velocity
MV: Mean velocity
LAD: Left anterior descending
RCA: Right coronary artery
WE: Water-excitation
FE: Fat-excitation
ICC: Intraclass correlation coefficient
R^2^: Coefficient of determination
